# Internal Migration and Mortality: The Case of Finland

**DOI:** 10.4137/EHI.S831

**Published:** 2008-06-13

**Authors:** Jan Saarela, Fjalar Finnäs

**Affiliations:** 1 Åbo Akademi University, Finland; 2 Åbo Akademi University, Finland

**Keywords:** internal migration, mortality, health selection, birth region, population data, Finland

## Abstract

In light of possibilities and limitations of data from the Finnish population register, and the general demographic development of Finland, this paper illuminates the complex interrelation between internal migration and mortality. We explore the roles played by health selection, birth region, and migration as a potentially harmful event. A five per cent random sample from a longitudinal data file that contains deaths for a period of 24 years is used. The focus is on people aged 40–59 years living in Southern Finland, who are defined by birth region and time since immigration. We find some indications of a healthy-migrant effect, but also that migrants may have integration difficulties or that they are negatively selected with regard to health behaviours and lifestyles. In line with previous studies on Finland, birth region is found to be a very decisive mortality determinant.

## Introduction

It is commonly known that population movements may influence the spatial distribution of health, implying that variation in mortality rates across subgroups of any regional population may be interrelated with population shifts that have occurred within the country. This issue appears partly overlooked in the academic and public debate on increased polarisation of morbidity and mortality rates within Europe (cf. Mackenbach, 2006). It could still be of utmost health promotion and policy concern, considering that there is regional variation in mortality rates and large internal migration flows within many countries.

Internal migration can affect mortality rates within a region due to three main reasons. First, migrants and non-migrants might be inherently different on health, saying that there is either positive or negative health selection in the out-migrant flow from the source region. Second, migration as an event may influence mortality risks because of difficulties in integrating into a new environment. This can raise stress levels or induce changes in health behaviours and lifestyles, which in turn may increase death risks. Third, within-region mortality variation might be an artefact of region-of-birth effects, particularly in the case when population movements within a country have been large. Such effects may occur if there is regional variation in hereditary factors, or in environmental circumstances at early childhood, which affect health at adult age.

Detailed insights into these issues require very comprehensive data. The longitudinal population register in Finland offers some opportunities. In light of the possibilities and limitations of data from this register, the purpose with the present paper is to use Finland as a case to enhance understanding of the interrelation between internal migration and mortality.

Next, we proceed by motivating why Finland is a useful country to study. The data are described in the subsequent chapter, together with a discussion on the methodological and theoretical considerations, which are quite complex and therefore require a lengthy exposition. The results of the empirical analyses are presented in the fourth chapter. The paper ends with some concluding comments.

## Internal Migration and Regional Mortality Differences in Finland

**Figure 1.**
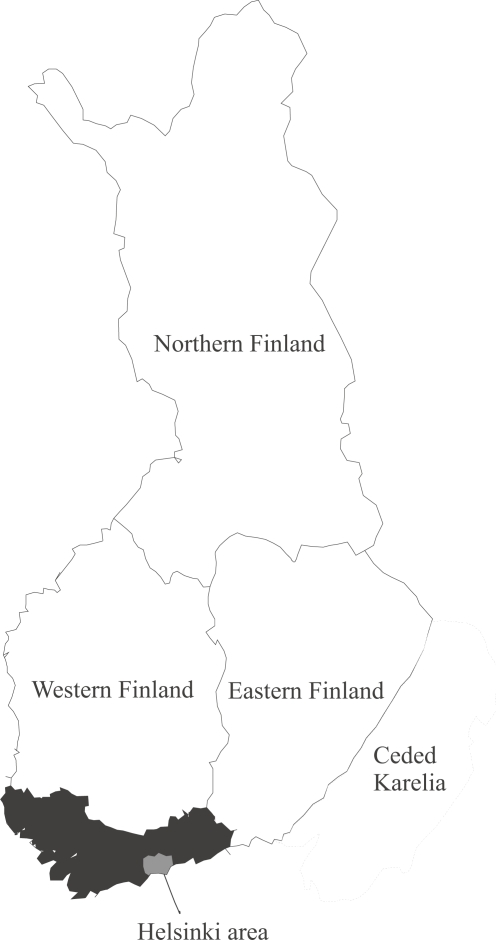
Map of Finland showing classification of region. **Note:** Southern Finland is represented by the shaded area. It includes Uusimaa, Itä-Uusimaa and Varsinais-Suomi. The Helsinki area consists of the municipalities Helsinki, Espoo, Vantaa and Kauniainen.

Not only the population register, but also the general demographic development of Finland makes the country a useful case for research on internal migration and mortality. Migration within the country has been substantial and predominantly in one direction. Since the process of modernisation started in the mid-nineteenth century there has been a gradual concentration of the population toward the South, which currently accounts for more than one third of the country’s total population (see Fig. 1). In the 1930s and after the Second World War the pace of this population shift was remarkably high, as the nation experienced the fastest industrialisation process in Europe. There was also a substantial increase in birth rates in the late 1940s, particularly in Eastern and Northern Finland, and many people in this baby boom generation subsequently moved to Southern Finland (Myrskylä, 1978; Korkiasaari and Söderling, 1994).

From the researcher’s point of view this migration pattern is very appealing, because mortality rates in Finland, both historical and present, tend to increase in the South-West to North-East direction (Pitkänen et al. 2000; Saarela and Finnäs, forthcoming), i.e. practically in the opposite direction to the main migration flows that have gone from high-mortality to low-mortality areas. An implication is that the current population in Southern Finland is very heterogeneous in terms of geographical extraction. This heterogeneity is relevant to bear in mind when considering that people who originate from high-mortality regions tend to have relatively high death rates also if moving elsewhere (Saarela and Finnäs, 2005; 2006; forthcoming). In 2000, less than 55 per cent of the population aged 40–59 years in Southern Finland were born there, a fifth in Eastern Finland and in Western Finland, respectively, and just over five per cent in Northern Finland. Migration from abroad has been marginal until the most recent years (Statistics Finland, 2008a).

Several studies have shown that birth region is an important predictor of adult mortality in Finland (Saarela and Finnäs, 2005; 2006; forthcoming). A question to be solved still is whether that effect might have to do with migration as a potentially harmful event. Saarela and Finnäs (2006) find that mortality rates of internal migrants are higher than of non-migrants. Since people in that study were aged over 60 years, and evidently observed several decades after having migrated, the results cannot tell us whether the event of migration influences mortality, however. Here we attempt to complement our knowledge on that point.

Since migration flows predominantly have gone in one direction—toward Southern Finland—we focus on this specific region when we analyse the effect of birth region on mortality rates. To cast some light on the potential effect of migration as an event on the mortality risk, we crudely categorise internal migrants by how long they had lived in Southern Finland. The issue of health selection of migrants is explored by comparing migrants with non-migrants according to birth region.

## Data and Methodological Considerations

### The population register in Finland

The data come from the longitudinal population census file compiled by Statistics Finland, and consist of individual-level information from the population censuses of 1970, 1975, 1980, 1985, 1990, 1995, and 2000 (Statistics Finland, 2008b). We have access to a five per cent random sample that contains basic demographic and socioeconomic variables. For all persons who had died during the period 1971–2004, the sample has been complemented with information about the year of death and main cause of death. Due to the construction of the internal migration variable (see below), all deaths in the analysis have still occurred after 1980.

We focus on people aged 40–59 years, i.e. ages that are fairly close to those where internal migration rates are the largest. Due to a few number of deaths at young adulthood, people aged 40–59 years represent the youngest groups for whom it is practically possible to perform rigorous analyses. Deaths that can be directly related to diseases are separated from other causes of death, which hereafter are called non-diseases (see Table 1).

As the total number of deaths is 1,318 in men and 622 in women, analysis with a more detailed categorisation of causes of death was not possible. In the ages under study, deaths due to non-diseases account for one third of all deaths in men and one fourth in women. The covariates used represent each person’s educational level and family situation. The latter was constructed on basis of civil status and family type. Since there is information about year of birth and year of death, we can calculate risk times and death risks at the single-year level.

As a complement to the data described above, we include an identically constructed 50 per cent random sample of the Swedish-speaking population in Finland. These individuals can in the population registers be separated from those in the Finnish-speaking majority on basis of their unique mother tongue. The Swedish speakers amount to barely six per cent of the country’s total population, and ten per cent of the population in Southern Finland. It is well documented that they have lower death rates and lower internal migration rates than Finnish speakers (Koskinen and Martelin, 2003; Saarela and Finnäs, 2006). They are included as a separate group in the analysis, in order to give a more enhanced picture of the situation in Southern Finland, and to help in interpreting the role played by factors associated with birth region. The two samples are analysed simultaneously by weighting the observations according to each sample proportion.

### Birth region and time since migration

Based on information about birth region and current region of residence, individuals are classified as migrants and non-migrants (the data do not contain explicit information about moves). Migrants are further crudely categorised according to how long they had lived in the destination area. The new variable consequently accounts for whether a person is a migrant, time since immigration, and geographic ancestry in terms of birth region.

Our attention is set at migration in a more long-term perspective, as some of the people who had migrated internally and returned to the source region within five years cannot be classified as migrants. We focus on long-distance migration within the country, as defined by moves between the four larger regions given in Figure 1. Confidentiality reasons restrict us from using very detailed spatial areas, but the present regional categorisation reflects geographic mortality differentials in Finland very well (cf. Saarela and Finnäs, 2005; 2006; 2008; forthcoming).

Since the main migration flows have gone in the direction of Southern Finland, we are particularly interested in the population currently residing in that area. Persons who were not born there are categorised according to whether they had lived at least ten years, or less than ten years, in the area.

Besides serving as a crude indicator for hereditary factors and circumstances at early childhood, birth region might also reflect similarity with the destination area in terms of aspects such as culture, climate and population density. People who originate from Western Finland would then have smaller difficulties in integrating into the environment of Southern Finland than those from, for example, Northern Finland.

In addition to these internal migrants, there is a category of people who were born in areas that were ceded to the Soviet Union because of the Second World War. These Karelians represent people restrained from moving back to their region of birth, and thus give some information about the consequences of internal displacement on mortality in the very long-term perspective. The Paris peace treaty implied that 430,000 persons or 11 per cent of Finland’s population were permanently evacuated elsewhere in the country. In the year 2000, 40 per cent of all the Karelians born 1921–1944 were living in Southern Finland, where they accounted for five per cent of the corresponding cohorts.

Swedish speakers in Finland, who live concentrated on the Southern and Western coastline, have always had much lower internal migration rates than Finnish speakers. Approximately 90 per cent of the Swedish speakers in Southern Finland were consequently also born there. The relatively few Swedish-speaking residents of Southern Finland who were born outside the area are not included in the analysis. The categorisation as discussed above consequently applies only to Finnish speakers. As categorised here, Swedish speakers will therefore not explicitly complement the picture about internal migration, but has the potential of illustrating the effect of a basic personal characteristic that is comparable to birth region.

With this classification, the variable that combines birth region and time since immigration consist of nine mutually exclusive categories (see Table A1 in the Appendix).

To explore whether the internal migrants who have settled in Southern Finland constitute a selected group on health, we compare them also (in separate models) with people in the source regions who have not migrated.

### Covariates and main causes of death

In case migration is a stressful event that affects persons’ health and raise their mortality levels (cf. Ben-Sira, 1997), one can argue that the effect should be stronger for more recent migrants. This is because they have had less time than migrants with long durations to adapt to the new environment, and any mechanisms that mediate and moderate presumed negative effects of stressors have not had equally much time to take effect (cf. Wheaton, 1985; Ensel and Lin, 1991).

If there is a healthy-migrant effect, as often argued in the international literature (Marmot et al. 1984; Friis et al. 1998), it should optimally be analysed and observed at the time point of migration. This approach is not fully accomplishable with present data, but we can still obtain some insight by observing death rates of more recent migrants, and particularly those for disease-related mortality, as such deaths proxy persons’ latent health condition.

In the analyses, we use a dummy variable that distinguish people who live in the Helsinki area, because they, similar to people in many other metropolitan regions, have relatively high death risks (Valkonen and Kauppinen, 2001). This feature is particularly prominent for non-disease mortality in women, and obviously reflects environmental factors prevalent in large cities (cf. Galea et al. 2005).

Factors associated with mortality interrelate highly also with migration rates (Blane et al. 1993; Davey Smith, 1996). Socioeconomic status as given by persons’ educational level is obviously one of the important variables in this respect, as it reflects selection and sorting on both health and migration intensity. To account for any confounding effects, educational level is used also here. It consists of five different categories that range from basic to higher-degree tertiary education.

If internal migrants have integration difficulties that affect their mortality risks, such problems in adapting are likely to correlate also with other characteristics that measure social capabilities. The most evident factor on this concern is family situation. Here, we use a variable that measures not only present status, but incorporates also changes in terms of partnership formation and dissolution. Thus, the variable will reflect capabilities of adapting to alterations of the family situation. As constructed here it is time-varying and gives changes in the family position during past ten years, consisting of the mutually exclusive categories of persons (1) presently with partner, never separated, (2) separated, with new partner, (3) separated, without new partner, and (4) never with partner. What is unusual in an international perspective is that we can consider not only marriages as partnerships, but also consensual unions.

In case deficiencies in social capabilities underlie elevated mortality risks of internal migrants, family situation will have a larger impact on non-disease mortality than on disease mortality, because the former are closer linked to individual behaviours and lifestyles, which in turn correlate with probabilities of partnership formation and dissolution. We also expect that recent migrants’ relative mortality risks are reduced more than less-recent migrants’ when account is taken for this variable, as integration problems tend to decrease over time. Since partnership does not only protect against poor health but selects the more healthy people in the population, it is still evident that family situation must be interrelated also with disease mortality.

The variable distributions are, together with those for birth region and time since immigration, given in Table A1 in the Appendix.

The results are consistently presented in the form of risk ratios for death, so that the mortality risk in each subgroup of interest is compared with the mortality risk in a reference category. Since our emphasis lies on overall patterns that can be observed from the data, we are not overly concerned with the statistical power of single parameters. For the sake of being comprehensive, we still provide 95 per cent confidence intervals for the estimates. Separate analyses are undertaken for men and women.

## Results

To begin with, we look at the results of some more general specifications based on death risks by region of birth for the population of Southern Finland, irrespective of how long the immigrants have lived in the area (Table 1). These rates are standardised for age and calendar year.

Birth region has a substantial impact on death rates also in these ages. As compared with men born in Southern Finland (regional natives), the death risk of men born in Northern Finland is 48 per cent higher. For men born in Eastern Finland and Ceded Karelia, the difference is almost 20 per cent. The mortality level of men born in Western Finland is practically the same as for those born in Southern Finland, which is in line with the general pattern for regional mortality differentials in Finland. Differences by birth region are most prominent for non-disease mortality, but can also be observed for disease mortality. As compared with Finnish speakers born in Southern Finland, Swedish speakers have ten per cent lower mortality risk of all causes, and more than twenty per cent lower risk of non-disease mortality.

The results for women must be considered in light of the relatively few deaths, and cannot be interpreted in an equally clear-cut manner. Unlike the case for men, women born in Western Finland have the lowest relative death rates, whereas those born in Northern Finland are at approximately the same levels as regional natives. Similar to the situation in men, Swedish-speaking women have relatively low death rates, particularly of non-diseases.

Next, we check if the picture remains the same when account is taken for migration duration and the covariates. This approach is accomplished by separating migrants into crude categories according to time since immigration, and by successively including variables for place of residence (whether the person lives in the metropolitan area), educational level, and family situation (Table 2 and Table 3).

Education is a very decisive determinant of mortality, as death risks of both diseases and non-diseases decrease notably with persons’ educational level. The variable has still a marginal impact in terms of reducing variation in mortality across birth regions.

Family situation also correlates strongly with mortality. Persons who live in a stable union have the lowest death risks. Variation in mortality by family situation, and particularly in non-disease mortality, is larger among men than among women. Separated men without a new partner, for example, have a death risk that is fourfold that of men who live in stable unions. Unlike the case with education, the estimated effects of the other variables are reduced notably when family situation is included into the models. This reduction is particularly prominent for non-disease mortality of recent immigrants, which reflects that they to a greater extent than non-migrants live in unstable unions.

Both men and women who live in the metropolitan area have elevated death risks. For non-disease mortality, it is approximately 40 per cent higher than that of persons living elsewhere in Southern Finland.

A general tendency concerning time since immigration is that disease mortality is lower for recent immigrants than for less recent immigrants, whereas non-disease mortality is higher. This suggests that migrants are in a good health position at the time of the move, and that unbeneficial consequences of birth region and/or of the new environment take effect in a longer-term perspective. For disease mortality in women, it is difficult to see any obvious pattern. Non-disease mortality is still clearly elevated among women categorised as recent immigrants.

A fair picture of how birth region influences mortality must incorporate the question of whether migrants are selected on health. We analyse this issue by comparing mortality levels of immigrants in Southern Finland with those for people who did not migrate from the source regions. Here we fit models separately by birth region, i.e. for people born in Western Finland, Northern Finland, and Eastern Finland, respectively. Similar to the procedure described above, covariates are successively included. Only the parameters for the variable that reflects migrant status are displayed in the summary of the results (Table 4).

Migrants in subgroups that have higher death rates than regional natives in Southern Finland tend to have higher death rates also as compared with people who had not migrated from the source regions. This suggests that, to some extent there has been negative health selection. However, the sign and size of such potential selection tends to differ across source regions and sexes. A completely unambiguous interpretation is therefore difficult to put forward, but some specific observations are still essential to highlight.

Men born in Western Finland tend to be positively selected on health, as indicated by the parameter for disease mortality of migrants with shorter duration. This might also explain why they, in relation to regional natives in Southern Finland, have so low rates of disease mortality (see Table 2). For women, similar positive health selection appears to have occurred for those who migrated from Eastern Finland, and they also have relatively modest disease-mortality rates as compared with regional natives in Southern Finland.

It is still important to note that migrants’ death rates as compared with regional natives’ are substantially lower than as compared with those of people who had not migrated from the source regions, and that this pattern is particularly emphasised for non-disease mortality. Migrants’ mortality risks are consequently affected also by the new environment, either directly because of exposure to hazards, or indirectly due to changes in health behaviours and lifestyles.

## Concluding Comments

The current population of Southern Finland, which has been of particular interest here due to the country’s general population development, amounts to almost two million persons. With a five per cent random sample, we could observe deaths during a 24-years period. In spite of these beneficial underpinnings, the number of deaths at young adulthood, i.e. in ages where people are most mobile, was still so few that a rigorous analysis of how death rates relate to internal migration was not possible. We therefore had to focus on people aged 40–59 years, and to distinguish only between disease mortality and non-disease mortality.

Hence, in spite of the data’s potentials in terms of total size, reliability and national coverage, they have some obvious limitations when it comes to studying the interrelation between internal migration and mortality. Future research on Finland, or corresponding cases, could therefore potentially make use of broader health measures than mortality, such as disability pensions or morbidity-based measures based on patient records. This would facilitate studies on younger ages as well. Other issues of potential importance is whether short-distance and short-term migration impact on health. Particularly the role played by the local environment seems to be of great concern, as presently illustrated by the elevated mortality risk of people who live in the Helsinki metropolitan area.

The general idea of a “healthy-migrant effect” says that migrants constitute groups of people with inherently better health than people who remain in the source regions. If that manifests in death rates, it should be observed as a relatively low incidence of disease-mortality close to the time point of migration, as partly illustrated by men in our data. However, migrants may also to some extent be people with unhealthy behaviours and hazardous lifestyles, and those with difficulties in integrating into the new environment, as illustrated by the markedly higher risks of non-disease mortality in women. Our findings consequently point at the importance of observing deaths by their main cause.

Even if we control for covariates that correlate highly with mortality risks, there remains a very strong effect of persons’ birth region. This feature is particularly prominent in men, which is natural from the point of view that regional mortality differentials are greater in men than in women. The low relative mortality rates of Swedish speakers in Finland can also be interpreted in a similar manner, i.e. as aspects affected not only by current environmental conditions or the level of integration, but also by factors that take effect before or at early childhood.

Many studies have found an elevated prevalence of stress symptoms among migrants (Noh and Avison, 1996; Harrison et al. 1997; Eaton and Garrison, 1992; Porter and Haslam, 2001; Hwang et al. 2007). A subgroup of particular interest in this respect consists of people born in areas that were ceded to the Soviet Union because of the Second World War. These people have been restrained from moving back to their source region, and thus have the potential of providing some insight into the issue of whether war-related migration impacts negatively on mortality in the very long term. This appears to be the case for men in our data, but not for women. We believe, however, that alternative model specifications and further data elaboration are needed before we can state something more conclusive on this matter. A primary goal for future research of ours is to analyse mortality rates of these internally displaced persons in detail.

Hence, the findings of our analyses lie within the spirit of much of the previous research in the area (Brimblecombe et al. 1999; 2000; Wannamethee et al. 2002; Connolly and O’Reilly, 2007). As migration postulates changes in persons’ living conditions in a multitude of ways, its interrelation with mortality is also multifaceted. On the one hand, migration intentions can correlate with health, and these intentions are generally driven by expectations of improved living standards that promote health and lower mortality risks. On the other hand, migration inevitably means changes in the social environment in which individuals live, and such changes might deteriorate health and increase the death risk. When additionally considering that death rates may depend on persons’ region of birth, the multidimensionality renders it not possible to expect an unambiguous solution to the migration-mortality interrelation.

## Figures and Tables

**Table 1.** Relative mortality differentials by birth region and sex, people aged 40–59 years residing in Southern Finland.

	**All causes**	**Diseases**	**Non-diseases**
Men by birth region			
Southern Finland (Finnish speaker)	1	1	1
Western Finland	0.99 (0.85–1.15)	0.99 (0.82–1.18)	1.01 (0.78–1.31)
Northern Finland	1.48 (1.22–1.81)	1.42 (1.11–1.83)	1.61 (1.16–2.25)
Eastern Finland	1.16 (1.00–1.35)	1.04 (0.86–1.26)	1.43 (1.12–1.83)
Ceded Karelia	1.20 (0.99–1.45)	1.19 (0.95–1.48)	1.25 (0.86–1.81)
Southern Finland (Swedish speaker)	0.90 (0.82–1.00)	0.95 (0.85–1.07)	0.77 (0.65–0.93)
Women by birth region			
Southern Finland (Finnish speaker)	1	1	1
Western Finland	0.74 (0.59–0.93)	0.66 (0.50–0.87)	0.95 (0.64–1.41)
Northern Finland	0.98 (0.71–1.33)	0.86 (0.58–1.26)	1.28 (0.75–2.17)
Eastern Finland	1.19 (0.98–1.46)	1.16 (0.92–1.47)	1.29 (0.89–1.86)
Ceded Karelia	1.03 (0.77–1.39)	1.00 (0.71–1.42)	1.14 (0.63–2.09)
Southern Finland (Swedish speaker)	0.81 (0.70–0.93)	0.87 (0.74–1.03)	0.61 (0.45–0.82)

The risk ratios account for effects of age and time period.

Numbers within parentheses give 95% confidence intervals.

Causes of death follow Statistics Finland’s 54-category short list of causes of death, which follows the 10th revision of the International Classification of Diseases (ICD-10). Diseases as referred to here correspond with codes 1–39 and non-diseases with codes 40–54 (Statistics Finland, 2008c).

Diseases amount to 70.2 per cent of all deaths in men and 76.7 in women.

Within non-diseases, alcohol-related causes (diseases and poisoning) amount to 36.1 per cent of all deaths in men, Suicides to 20.0, and External causes excluding suicides to 43.9. Corresponding numbers in women are 30.3, 23.0 and 46.7 per cent, respectively.

**Table 2.** Risk ratios for death in Southern Finland, men aged 40–59 years.

	**All causes**				**Diseases**				**Non-diseases**			
Birth region and time since immigration												
Southern Finland (Finnish speaker)	1	1	1		1	1	1		1	1	1	
Western Finland, immigrated 10+ years ago	0.99	1.02	1.04	(0.89–1.21)	1.01	1.03	1.05	(0.87–1.26)	0.96	1.01	1.04	(0.80–1.36)
Western Finland, immigrated <10 years ago	0.80	0.86	0.74	(0.43–1.25)	0.64	0.68	0.59	(0.28–1.26)	1.06	1.15	0.96	(0.45–2.05)
Northern Finland, immigrated 10+ years ago	1.45	1.45	1.43	(1.16–1.76)	1.42	1.42	1.40	(1.08–1.82)	1.51	1.52	1.50	(1.05–2.14)
Northern Finland, immigrated <10 years ago	1.62	1.69	1.43	(0.83–2.49)	1.39	1.45	1.25	(0.59–2.64)	1.99	2.09	1.73	(0.76–3.90)
Eastern Finland, immigrated 10+ years ago	1.13	1.12	1.11	(0.95–1.30)	1.03	1.03	1.02	(0.84–1.23)	1.34	1.33	1.32	(1.03–1.70)
Eastern Finland, immigrated <10 years ago	1.16	1.16	0.97	(0.58–1.61)	1.12	1.11	0.95	(0.49–1.85)	1.22	1.22	0.98	(0.43–2.22)
Ceded Karelia	1.19	1.19	1.20	(0.99–1.45)	1.19	1.19	1.19	(0.95–1.49)	1.23	1.23	1.26	(0.87–1.83)
Southern Finland (Swedish speaker)	0.90	0.92	0.93	(0.84–1.03)	0.95	0.97	0.98	(0.87–1.10)	0.78	0.81	0.82	(0.68–0.98)
Lives in Helsinki area												
No	1	1	1		1	1	1		1	1	1	
Yes	1.12	1.23	1.14	(1.06–1.24)	1.03	1.11	1.05	(0.96–1.16)	1.37	1.51	1.36	(1.18–1.57)
Educational level												
Primary		1	1			1	1			1	1	
Secondary		0.79	0.81	(0.73–0.89)		0.79	0.80	(0.71–0.91)		0.79	0.80	(0.67–0.95)
Lowest tertiary		0.68	0.75	(0.65–0.86)		0.73	0.80	(0.67–0.94)		0.58	0.66	(0.51–0.84)
Lower-degree tertiary		0.54	0.62	(0.52–0.74)		0.60	0.67	(0.55–0.83)		0.44	0.53	(0.38–0.74)
Higher-degree tertiary		0.36	0.41	(0.34–0.50)		0.39	0.43	(0.35–0.54)		0.31	0.38	(0.27–0.53)
Family situation (past 10 years)												
With partner, never separated			1				1				1	
Separated, with new partner			1.51	(1.29–1.77)			1.44	(1.19–1.74)			1.75	(1.31–2.33)
Separated, without new partner			2.77	(2.53–3.03)			2.28	(2.04–2.55)			4.09	(3.49–4.81)
Never with partner			2.37	(2.12–2.65)			2.19	(1.92–2.50)			2.88	(2.34–3.53)

Estimates for the effects of age and time period are not displayed.

Numbers within parentheses give 95% confidence intervals.

Number of individuals in the unweighted sample is 40,610, number of person years 478,396, number of deaths due to diseases 1,870, and number of deaths due to non-diseases 795.

Immigrants as categorised here correspond with out-migrants as referred to in Table 4.

**Table 3.** Risk ratios for death in Southern Finland, women aged 40–59 years.

	**Causes**				**Diseases**				**Non-Diseases**			
Birth region and time since immigration												
Southern Finland (Finnish speaker)	1	1	1		1	1	1		1	1	1	
Western Finland, immigrated 10+ years ago	0.70	0.72	0.73	(0.58–0.92)	0.61	0.63	0.64	(0.48–0.85)	0.91	0.96	0.98	(0.65–1.46)
Western Finland, immigrated <10 years ago	1.00	1.08	1.01	(0.50–2.05)	1.07	1.13	1.09	(0.48–2.46)	0.84	0.96	0.85	(0.21–3.49)
Northern Finland, immigrated 10+ years ago	0.90	0.92	0.92	(0.67–1.28)	0.81	0.82	0.82	(0.55–1.23)	1.15	1.20	1.21	(0.69–2.11)
Northern Finland, immigrated <10 years ago	1.35	1.44	1.32	(0.54–3.20)	1.13	1.18	1.10	(0.35–3.45)	1.86	2.17	1.90	(0.46–7.75)
Eastern Finland, immigrated 10+ years ago	1.11	1.10	1.11	(0.90–1.36)	1.13	1.11	1.12	(0.88–1.42)	1.09	1.08	1.09	(0.74–1.61)
Eastern Finland, immigrated <10 years ago	1.38	1.49	1.38	(0.73–2.60)	0.79	0.84	0.81	(0.30–2.17)	2.78	3.08	2.67	(1.15–6.16)
Ceded Karelia	1.00	1.00	1.00	(0.74–1.34)	0.98	0.98	0.98	(0.69–1.38)	1.09	1.09	1.09	(0.60–1.99)
Southern Finland (Swedish speaker)	0.80	0.81	0.82	(0.71–0.95)	0.87	0.88	0.89	(0.75–1.05)	0.61	0.62	0.63	(0.47–0.85)
Lives in Helsinki area												
No	1	1	1		1	1	1		1	1	1	
Yes	1.24	1.31	1.23	(1.10–1.38)	1.19	1.25	1.18	(1.04–1.34)	1.39	1.48	1.38	(1.09–1.74)
Educational level												
Primary		1	1			1	1			1	1	
Secondary		0.62	0.61	(0.53–0.71)		0.64	0.63	(0.54–0.74)		0.55	0.56	(0.42–0.76)
Lowest tertiary		0.48	0.49	(0.39–0.61)		0.61	0.60	(0.46–0.77)		0.25	0.26	(0.16–0.44)
Lower-degree tertiary		0.59	0.59	(0.47–0.76)		0.60	0.59	(0.45–0.79)		0.56	0.59	(0.38–0.94)
Higher-degree tertiary		0.42	0.42	(0.30–0.58)		0.41	0.40	(0.26–0.59)		0.43	0.45	(0.25–0.82)
Family situation (past 10 years)												
With partner, never separated			1				1				1	
Separated, with new partner			1.50	(1.20–1.88)			1.13	(0.85–1.50)			2.39	(1.62–3.51)
Separated, without new partner			1.63	(1.43–1.85)			1.50	(1.30–1.75)			1.99	(1.53–2.59)
Never with partner			1.79	(1.52–2.11)			1.89	(1.57–2.26)			1.50	(1.04–2.18)

Estimates for the effects of age and time period are not displayed.

Numbers within parentheses give 95% confidence intervals.

Number of individuals in the unweighted sample is 41,309, number of person years 495,192, number of deaths due to diseases 988, and number of deaths due to non-diseases 300.

Immigrants as categorised here correspond with out-migrants as referred to in Table 4.

**Table 4.** Risk ratios for death of out-migrants (to Southern Finland) in relation to non-migrants by birth region and sex, persons aged 40–59 years.

	**All causes**			**Diseases**			**Non-diseases**		
	**Model 1**	**Model 2**	**Model 3**	**Model 1**	**Model 2**	**Model 3**	**Model 1**	**Model 2**	**Model 3**
MEN									
Western Finland, Non-migrant	1	1	1	1	1	1	1	1	1
Out-migrated, 10+ years ago	0.96	1.07	1.06 (0.92–1.22)	0.98	1.08	1.08 (0.91–1.28)	0.93	1.06	1.04 (0.81–1.33)
Out-migrated, <10 years ago	0.76	0.87	0.74 (0.43–1.25)	0.61	0.69	0.62 (0.29–1.30)	1.00	1.17	0.90 (0.43–1.92)
Northern Finland, Non-migrant	1	1	1	1	1	1	1	1	1
Out-migrated, 10+ years ago	1.28	1.39	1.38 (1.11–1.70)	1.23	1.32	1.30 (0.99–1.69)	1.41	1.58	1.57 (1.09–2.26)
Out-migrated, <10 years ago	1.41	1.60	1.38 (0.79–2.40)	1.19	1.32	1.15 (0.54–2.45)	1.80	2.15	1.80 (0.79–4.10)
Eastern Finland, Non-migrant	1	1	1	1	1	1	1	1	1
Out-migrated, 10+ years ago	0.90	1.00	0.97 (0.84–1.12)	0.87	0.96	0.94 (0.78–1.13)	0.97	1.08	1.04 (0.82–1.31)
Out-migrated, <10 years ago	0.91	0.99	0.81 (0.48–1.35)	0.94	1.02	0.87 (0.45–1.68)	0.85	0.95	0.73 (0.33–1.65)
WOMEN									
Western Finland, Non-migrant	1	1	1	1	1	1	1	1	1
Out-migrated, 10+ years ago	0.76	0.79	0.77 (0.62–0.96)	0.61	0.63	0.62 (0.48–0.82)	1.28	1.38	1.35 (0.92–1.98)
Out-migrated, <10 years ago	1.05	1.11	1.04 (0.52–2.10)	1.05	1.09	1.03 (0.46–2.32)	1.07	1.19	1.09 (0.27–4.45)
Northern Finland, Non-migrant	1	1	1	1	1	1	1	1	1
Out-migrated, 10+ years ago	0.90	0.96	0.91 (0.65–1.27)	0.69	0.73	0.70 (0.46–1.05)	1.99	2.18	1.96 (1.06–3.63)
Out-migrated, <10 years ago	1.36	1.53	1.37 (0.56–3.33)	0.99	1.09	1.02 (0.32–3.21)	3.17	3.86	2.90 (0.69–12.2)
Eastern Finland, Non-migrant	1	1	1	1	1	1	1	1	1
Out-migrated, 10+ years ago	1.30	1.35	1.26 (1.04–1.54)	1.25	1.28	1.21 (0.96–1.53)	1.51	1.57	1.44 (0.97–2.14)
Out-migrated, <10 years ago	1.56	1.73	1.44 (0.76–2.72)	0.84	0.92	0.79 (0.29–2.14)	3.67	4.22	3.22 (1.38–7.53)

Model 1 accounts for age and time period.

Model 2 accounts for age, time period and educational level.

Model 3 accounts for age, time period, educational level and family situation.

Numbers within parentheses give 95% confidence intervals.

People born in Ceded Karelia and Swedish speakers are excluded.

Out-migrants as categorised here correspond with immigrants as referred to in Tables 2 and 3.

## Appendix

**Table A1.** Number of deaths and total risk time by variable category.

	**Men**			**Women**		
	**# deaths diseases**	**# deaths non-diseases**	**Person years**	**# deaths diseases**	**# deaths non-diseases**	**Person years**
Birth region and time since immigration						
Southern Finland (Finnish speaker)	371	179	104,028	185	70	102,061
Western Finland, immigrated 10+ years ago	168	76	43,450	63	36	53,530
Western Finland, immigrated <10 years ago	7	7	3,901	6	2	3,699
Northern Finland, immigrated 10+ years ago	67	37	13,872	27	15	17,701
Northern Finland, immigrated <10 years ago	7	6	1,776	3	2	1,647
Eastern Finland, immigrated 10+ years ago	147	96	38,135	109	41	48,916
Eastern Finland, immigrated <10 years ago	9	6	2,849	4	6	3,229
Ceded Karelia	101	34	14,168	40	13	14,131
Southern Finland (Swedish speaker)	993	354	256,217	551	115	250,278
Lives in Helsinki area						
No	1,058	383	271,794	510	129	264,975
Yes	812	412	206,602	478	171	230,217
Educational level						
Primary	1,169	450	215,393	642	189	237,820
Secondary	357	195	114,859	201	62	124,020
Lowest tertiary	160	73	57,833	67	16	64,711
Lower-degree tertiary	101	37	42,551	53	21	39,052
Higher-degree tertiary	83	40	47,760	25	12	29,589
Family situation (past 10 years)						
With partner, never separated	1,003	313	328,214	511	130	310,029
Separated, with new partner	122	55	31,673	54	33	28,379
Separated, without new partner	458	294	72,089	271	101	107,258
Never with partner	287	133	46,420	152	36	49,526

The description is for the unweighted sample of people aged 40–59 years who reside in Southern Finland.
